# Identifying Sources of Children’s Consumption of Junk Food in Boston After-School Programs, April–May 2011

**DOI:** 10.5888/pcd11.140301

**Published:** 2014-11-20

**Authors:** Erica L. Kenney, S. Bryn Austin, Angie L. Cradock, Catherine M. Giles, Rebekka M. Lee, Kirsten K. Davison, Steven L. Gortmaker

**Affiliations:** Author Affiliations: S. Bryn Austin, Angie L. Cradock, Catherine M. Giles, Rebekka M. Lee, Kirsten K. Davison, Steven L. Gortmaker, Harvard School of Public Health, Boston, Massachusetts.

## Abstract

**Introduction:**

Little is known about how the nutrition environment in after-school settings may affect children’s dietary intake. We measured the nutritional quality of after-school snacks provided by programs participating in the National School Lunch Program or the Child and Adult Care Food Program and compared them with snacks brought from home or purchased elsewhere (nonprogram snacks). We quantified the effect of nonprogram snacks on the dietary intake of children who also received program-provided snacks during after-school time. Our study objective was to determine how different sources of snacks affect children’s snack consumption in after-school settings.

**Methods:**

We recorded snacks served to and brought in by 298 children in 18 after-school programs in Boston, Massachusetts, on 5 program days in April and May 2011. We measured children’s snack consumption on 2 program days using a validated observation protocol. We then calculated within-child change-in-change models to estimate the effect of nonprogram snacks on children’s dietary intake after school.

**Results:**

Nonprogram snacks contained more sugary beverages and candy than program-provided snacks. Having a nonprogram snack was associated with significantly higher consumption of total calories (+114.7 kcal, *P* < .001), sugar-sweetened beverages (+0.5 oz, *P* = .01), desserts (+0.3 servings, *P* < .001), and foods with added sugars (+0.5 servings; *P* < .001) during the snack period.

**Conclusion:**

On days when children brought their own after-school snack, they consumed more salty and sugary foods and nearly twice as many calories than on days when they consumed only program-provided snacks. Policy strategies limiting nonprogram snacks or setting nutritional standards for them in after-school settings should be explored further as a way to promote child health.

## Introduction

Snacks are an important target area for improving children’s diets and potentially reducing childhood obesity (1). Most US children aged 2 to18 years snack regularly; snacks account for 27% of total calorie intake and are frequently composed of desserts, salty foods, and sugar-sweetened beverages (2), which increase the risk of obesity (3). Children consume fewer calories and feel more sated when their snacks are nutrient- and fiber-rich foods (4). Improving the healthfulness of children’s snacks could reduce excess energy intake and increase intake of healthier foods and beverages, such as fruits, vegetables, whole grains, and water.

After-school programs are a key setting for improving children’s snacks. These programs reach approximately 8.4 million school-aged children in the United States, or 15% of the school-aged population (5), and often serve at least 1 snack (program-provided snack) daily. Children in after-school programs may also consume snacks obtained elsewhere (nonprogram snacks*)*. Little is known about after-school snacks, although evidence suggests that program-provided snacks rarely include sugar-sweetened beverages (6–8), and programs that participate in the National School Lunch Program (NSLP) (www.fns.usda.gov/nslp/national-school-lunch-program-nslp) or Child and Adult Care Food Program (CACFP) (www.fns.usda.gov/cacfp/regulations) for federal snack reimbursement must adhere to basic nutrition standards (9). Even less is known about nonprogram snacks, which are not typically covered by standards (10).

In schools and child-care settings, foods and beverages obtained outside institutional meal programs may be less healthful. Meals provided by parents to children in child care centers do not meet children’s nutritional needs as well as program-provided meals do (11). Availability of competitive foods (ie, from school stores or vending machines) in kindergarten through 12th grade settings is associated with consumption of less healthful foods (12,13) and higher body mass index (BMI) *z* scores (14). Neither the effect of nonprogram snacks in after-school programs on children’s diet nor the nutritional quality of these snacks is known.

Our study objective was to determine how sources of snacks affect children’s snack consumption in after-school settings. The study aimed 1) to compare the nutritional quality of snacks provided by after-school programs participating in NSLP or CACFP with the quality of snacks brought by children and 2) to quantify the effect of these different sources of snacks on dietary intake after school. We hypothesized that program-provided snacks would be more healthful and that children would consume more total calories when a nonprogram snack was part of their snack.

## Methods

### Sample

This study used an observational, within-child, change-in-change design. Study participants were children attending after-school programs in Boston, Massachusetts, who were participating in follow-up data collection for the Out of School Nutrition and Physical Activity (OSNAP) intervention trial in April and May 2011 (15). Of 31 eligible programs, 20 programs were originally recruited to participate in the OSNAP intervention trial (10 intervention, 10 control). Eligible programs had to serve at least 39 children, operate from mid-October through June 1, and be willing to be randomized. All children attending participating programs were thereby eligible participants. Of the 1,231 children attending participating programs in April and May 2011, 564 agreed to participate in dietary intake assessment. Parents provided written consent, and children provided verbal consent. Because our study used a within-child design, the sample was restricted to children who were not absent on any study day. One entire program with 22 participants missed 1 study day; additionally, 177 participants were absent on 1 or both study days. Because our goal was to compare nonprogram snacks with program-provided snacks in programs participating in NSLP or CACFP, 1 program that did not participate in NSLP or CACFP was excluded. This resulted in a final sample of 18 programs and 298 children; participating children did not differ demographically from nonparticipating children. 

The study was originally powered to detect a difference of 0.2 servings per day of fruits and vegetables with 90% power. Each snack offered by the program or provided by participants was observed for 5 consecutive days, and children’s consumption of these snacks was observed on 2 days. The study was approved by the Harvard School of Public Health Committee on Human Subjects and the Boston Public Schools’ Department of Data and Accountability.

### Measures

*Child and program characteristics.* The age, sex, grade level, and race/ethnicity of each participating child were reported by parents on consent forms. The type of food service provider used was collected from school administrative records, and program enrollment was reported by after-school program directors. The program’s written nutrition policies were collected and coded by study staff; programs were categorized as having a written policy addressing nonprogram snacks if any written statements were found in their policy documents referring to limiting the foods children could bring.

*Program-provided and nonprogram snacks served.* On each of the 5 consecutive days of data collection, trained observers recorded the serving size, brand, and flavor of each food and beverage item served in program-provided and nonprogram snacks.

*Snack intake.* On 2 days of data collection, observers estimated by visual observation children’s dietary intake during after-school snack periods. Observers examined each child’s leftovers for each food and beverage, compared those with each food’s original serving size, and rated whether the child ate none, some, most, or all of each food. If a child had multiple servings of a food, consumption was rated for each serving. The amount consumed of each food for each serving was then calculated as 100% if rated as “all,” 66.7% if rated as “most,” 33.3% if rated as “some,” and 0% if rated as “none.” These estimation methods were found to be highly valid when compared with estimates of consumption derived from weighing children’s plates before and after consumption (E.L. Kenney, unpublished data, June 2013).

*Nutritional information.* Detailed nutrient and ingredient information was collected for each food item served using methods designed by Mozaffarian et al (6). Data for programs whose snacks were provided through Boston Public Schools’ Food and Nutrition Services were obtained directly from that agency, which is responsible for 80% of after-school snack programs. For other foods and beverages, data were obtained from manufacturers’ or grocery stores’ websites or from the US Department of Agriculture Nutrient Database (16). Foods and beverages were defined as containing added sugars if a caloric sweetener was one of the first 3 ingredients listed. Foods were defined as whole grain if the first ingredient was a whole grain. Sugar-sweetened beverages were defined as beverages with caloric sweeteners (excluding sweetened milk), including nondiet soda, fruit drinks containing less than 100% juice, lemonade, sweetened teas, energy drinks, or sports drinks. Salty snacks were defined as chips, pretzels, or salted crackers. Desserts were defined as pastries, cakes, cookies, puddings, or ice cream.

### Statistical analysis

To compare the nutritional quality of program-provided and nonprogram snacks served, we calculated on each of the 5 days of data collection the average total calories (kcal) and kilocalories from beverages served per snack; ounces of water, 100% juice, milk, and sugar-sweetened beverages served; and servings of fruits, vegetables, whole grains, foods with added sugars, desserts, candy, and salty snacks served to each child in program-provided and nonprogram snacks. A “serving” is a single serving as defined by a product’s nutrition label. For fruits and vegetables, serving size was determined by CACFP for snacks served to children. We then used paired *t* tests to compare program-provided with nonprogram snacks on each of these food and beverage categories within each program.

To evaluate whether the presence of a nonprogram snack affected a child’s consumption of total calories and the foods and beverages listed above during after-school program time, we examined within-child change in dietary intake during the after-school snack period on 2 observation days. Regression models evaluated how switching from having only a program-provided snack to having a nonprogram snack available affected a child’s intake of total calories and a range of food and beverages. This approach allowed each child to serve as his or her own control, removing the effect of potential time-stable confounders, such as socioeconomic status and body size, which may have resulted in confounding in a between-subjects model (17). By testing interaction terms in the models, we evaluated whether the effect of eating a nonprogram snack might vary according to the following: 1) a child’s age or sex, 2) the day of the week, 3) whether or not the program had a nutrition policy about the type of foods allowed in nonprogram snacks, and 4) whether or not the program participated in the OSNAP intervention. All statistical tests were 2-sided, with the level of statistical significance set to α = .05. All models were adjusted for clustering at the after-school program level using the PROC GLM procedure in SAS version 9.3 (Cary, North Carolina).

## Results

### Program and child characteristics

Most programs (61%) served snacks pre-prepared by an off-site food-service vendor (Table 1). Six programs served snacks prepared by an on-site school kitchen and one bought their snacks from the grocery store. Of the 18 programs, 10 (55.6%) had received the OSNAP intervention. Six programs (33.3%) had a written policy limiting the type of foods children could bring in from outside the snack program. The mean age of the children in the sample for analysis was 7.6 (SD, 1.8) years; 48.7% were girls. Most children were identified by their parents as black or Hispanic, with smaller groups of children identified as white, Asian American, multiracial, or another race/ethnicity.

**Table 1:** Baseline Characteristics of a Sample of Children (N = 298) and After-School Programs (N = 18), Study of Snack Consumption, Boston, Massachusetts, April–May 2011^a^

Characteristic	Value
**Child**	
**Age, mean (SD)**	**7.6 (1.8)**
**Female, n (%)**	**145 (48.7)**
**Race/ethnicity, n (%)**	
White, non-Hispanic	16 (6.6)
Black, non-Hispanic	97 (40.1)
Hispanic	96 (39.7)
Asian American	11 (4.6)
Multiracial	14 (5.6)
Other	8 (3.3)
**Consumed a nonprogram snack, day 1, n (%)**	**77 (25.8)**
**Consumed a nonprogram snack, day 2, n (%)**	**65 (21.8)**
**Program**	
**Sponsoring agency, n (%)**	
YMCA of Greater Boston	7 (38.9)
Boys and Girls Club	3 (16.7)
Community Center	4 (22.2)
School	4 (22.2)
**Food service operations, n (%)**	
Off-site snack vendor	11 (61.1)
On-site school kitchen	6 (33.3)
Independent	1 (5.6)
**Children enrolled per program, mean (SD)**	**64.5 (29.2)**
**Written program policy limits foods brought in from outside the snack program, n (%)**	**6 (33.3)**
**Received OSNAP intervention, n (%)**	**10 (55.6)**

Abbreviations: SD, standard deviation; OSNAP, Out of School Nutrition and Physical Activity intervention.

a Totals vary because of missing data

### Foods and beverages available in program-provided and nonprogram snacks

During the 5 days of observation, program-provided snacks included significantly more servings of milk (*P* = .003) and 100% juice (*P* < .001) than nonprogram snacks (Table 2). Sugar-sweetened beverages and candy were never available in program-provided snacks, whereas the daily mean volume of sugar-sweetened beverages brought in by children was 1.7 (SD, 1.5) ounces per child; candy was observed in nearly 1 of 10 nonprogram snacks. Program-provided snacks included significantly more servings of fruits (*P* = .002), vegetables (*P* < .001), whole grains (*P* = .02), and foods with added sugars (*P* = .003) than nonprogram snacks.

**Table 2:** Average Daily Values of Snack Foods Eaten by Children at After-School Programs (N = 18) in Boston, Massachusetts, April–May 2011

Food	Program-Provided Snacks, Mean (SD)	Nonprogram^a^ Snacks, Mean (SD)	*P* Value^b^
Total kcal	280.9 (129.9)	158.3 (54.1)	.002
Kcal from beverages	86.6 (54.5)	21.8 (16.0)	<.001
Water, oz	2.8 (2.7)	2.0 (1.5)	.22
100% juice, oz	3.2 (2.2)	0.2 (0.4)	<.001
Milk, oz	4.2 (4.4)	0.4 (1.3)	.003
Sugar-sweetened beverages, oz	0	1.7 (1.5)	<.001
Fruit, servings^c^	0.4 (0.2)	0.2 (0.2)	.002
Vegetables, servings^c^	0.2 (0.2)	0.002 (0.005)	<.001
Whole grains, servings^c^	0.4 (0.4)	0.1 (0.1)	.02
Foods with added sugars, servings^c^	1.0 (0.6)	0.5 (0.2)	.003
Dessert, servings^c^	0.2 (0.2)	0.2 (0.1)	.58
Candy, servings^c^	0	0.1 (0.1)	<.001
Salty snacks, servings^c^	0.5 (0.3)	0.4 (0.2)	.20

Abbreviations: SD, standard deviation.

a Nonprogram snacks are snacks that are not provided by the after-school program: children purchase them or bring them from home.

b *P* values are from paired *t *tests comparing program-provided snacks with nonprogram snacks in each after-school program; all tests were 2-sided.

c A “serving” is a single serving as defined by a product’s nutrition label. For fruits and vegetables, a serving size is determined by the Child and Adult Care Food Program for snacks served to children (http://www.fns.usda.gov/cacfp/regulations).

### Estimated effect of nonprogram snacks on dietary intake

When children had only a program-provided snack, they consumed an average of 130.3 (SD, 91.8) kcal; when they also had a nonprogram snack, children consumed an average of 275.9 (SD, 151.8) kcal (Table 3), a crude difference of 145.6 kcal. Independent of snack type, children ate fruits and vegetables infrequently. Candy and sugar-sweetened beverages were consumed only as nonprogram snacks.

**Table 3:** Average Calories in Snacks Consumed Over 2 Observation Days by Children (N = 298) in After-School Programs (N = 18) and Adjusted Change in Kcal Intake Associated With Nonprogram Snacks^a^, Boston, Massachusetts, April–May 2011

Food	Consumed Only Program-Provided Snack, Mean (SD)	Consumed Nonprogram Snack, Mean (SD)^b^	Adjusted Change In Intake (95% CI)^c^	*P* Value
Total kcal	130.3 (91.8)	275.9 (151.8)	+114.7 (85.1 to 144.3)	<.001
Beverages, kcal	38.3 (52.1)	45.4 (65.1)	+4.7 (−11.2 to 20.7)	.56
Water, oz	1.0 (2.2)	1.3 (4.0)	+0.4 (−0.2 to 1.1)	.19
100% juice, oz	1.9 (2.8)	1.4 (2.9)	−0.5 (−1.3 to 0.4)	.26
Milk, oz	0.5 (1.7)	0.6 (2.3)	+0.5 (0.01 to 1.1)	.05
Sugar-sweetened beverages, oz	0	1.1 (3.4)	+0.5 (0.1 to 0.9)	.01
Fruit, servings^d^	0.3 (0.8)	0.4 (0.6)	+0.2 (−0.1 to 0.4)	.24
Vegetables, servings	0.05 (0.2)	0.02 (0.1)	−0.05 (−0.1 to 0.01)	.09
Whole grains, servings	0.2 (0.4)	0.2 (0.4)	+0.08 (−0.04 to 0.2)	.20
Foods with added sugars, servings	0.4 (0.6)	1.0 (1.0)	+0.5 (0.3 to 0.7)	<.001
Dessert, servings	0.1 (0.4)	0.4 (0.6)	+0.3 (0.2 to 0.4)	<.001
Candy, servings	0	0.2 (0.5)	+0.2 (0.1 to 0.3)	<.001
Salty snacks, servings	0.3 (0.7)	0.7 (1.0)	+0.2 (−0.03 to 0.4)	.10

Abbreviations: SD, standard deviation; CI, confidence interval.

a Nonprogram snacks are snacks that are not provided by the after-school program: children purchase them or bring them from home.

b When children consumed a nonprogram snack, they could have also consumed some or all of a program-provided snack.

c Adjusted change estimates and *P* values are from fixed-effects regression models, which adjust for time-stable confounders. Models also took into account the clustered sampling design.

d A “serving” is a single serving as defined by a product’s nutrition label. For fruits and vegetables, a serving size is determined by the Child and Adult Care Food Program for snacks served to children (http://www.fns.usda.gov/cacfp/regulations).

In the within-child change-in-change analysis (Table 3), having a nonprogram snack was associated with significantly higher consumption of total calories during the after-school snack period (+114.7 kcal, 95% CI, 85.1–144.3) than when a child did not have a nonprogram snack. When a child consumed a nonprogram snack, he or she consumed, on average, an additional 0.5 oz of sugar-sweetened beverages (95% CI, 0.1–0.9), 0.3 servings of desserts (95% CI, 0.2–0.4), and 0.2 servings of candy (95% CI, 0.1–0.3) during the snack period. Notably, children ate an extra half-serving of foods with added sugars when they consumed a nonprogram snack (+0.5 servings, 95% CI, 0.3–0.7). There were no significant interactions between having a nonprogram snack and a child’s age or sex, a program’s nutrition policy, OSNAP intervention status, or the day of the week; therefore, these interaction terms were not included in the final models.

## Discussion

On days when children brought their own snack to the after-school program, they consumed, on average, an extra 115 kcal and more salty and sugary foods than when they consumed only a snack served by the after-school program. The children in this study did not substitute the snacks they brought for program-provided snacks. Rather, when both were available, they frequently consumed both, consuming nearly double the total calories and servings of foods with added sugars and triple the servings of desserts as when they consumed the program-provided snack alone. Additionally, children consumed an average of 1.1 oz of sugar-sweetened beverages per day when they had a nonprogram snack, and consumed no sugar-sweetened beverages when they had only the program-provided snack. Program-provided snacks that meet NSLP and CACFP standards may meet most children’s caloric needs alone for snacks. In this study, children eating only a program snack consumed an average of 130.3 kcal, within 133 kcal range of the average kcal per snack suggested by the Institute of Medicine for children aged 5 to 10 years (18).

Although older and larger children may need more calories to maintain their weight and healthy growth, the effect of a nonprogram snack was independent of age, suggesting that for some children the nonprogram snack may have resulted in excess calorie intake after school. A nationally representative study of the relationship between school food environments and children’s dietary behaviors found that higher availability of sugar-sweetened beverages and competitive foods in schools resulted in higher overall daily calorie intake (13), suggesting that children do not necessarily compensate for high calorie intake at school by eating less elsewhere. If the children in this study did not compensate by eating less or increasing energy expenditure, the extra calories they consumed could put them at risk for excess weight gain. It is estimated that an average daily excess intake of 110 to 165 kcal leads to the average weight gain observed among US children (19).

In addition to consuming more total calories, children also consumed less healthful foods when given the opportunity to consume a nonprogram snack, including more foods with added sugars, desserts, candy, salty snacks, and sugar-sweetened beverages. Sugar-sweetened beverages have been systematically linked with obesity (3), and a recent randomized controlled trial showed that lowering consumption of these beverages reduces weight gain by children and adolescents (20). National recommendations discourage excess consumption of foods with added sugars and refined carbohydrates because of their potential to displace nutrient-rich foods and their negative influence on blood glucose levels (21,22). Recent evidence suggests that consuming sugary foods and refined carbohydrates is associated with excess weight gain by adults (23), but the effects on children have been less studied. Frequent consumption of these foods and beverages during childhood could lead children to form and reinforce unhealthy eating habits that persist into adulthood (24).

These results also suggest a potential opportunity for after-school programs that provide snacks to make significant positive change in children’s diets through policy and environmental changes. After-school programs can promote access to healthy foods and beverages by instituting policies like those that have recently been adopted by several states and school districts to limit student access to competitive unhealthful foods (25). Schools could extend the new “Smart Snacks in School” standards for foods sold during the school day (26) so that the standard also applies to after-school programs. Programs could set clear nutritional standards for snacks provided from home or in vending machines, restrict vending machine access, or ban all nonprogram snacks from the after-school program. Such policy and environment changes could help ensure that the only choice for children during program hours is a healthful snack. Studies show that policies limiting the availability of snack foods during the regular school day can reduce children’s consumption of sugar-sweetened beverages and improve fruit and vegetable consumption (27,28). Recent evidence shows that changing from an environment with weak to strong competitive food policies is significantly associated with a reduction in adolescents’ BMI (25). Similar policies for after-school programs could have beneficial effects.

The use of a precise and reliable observation method to record all children’s dietary intake in the after-school setting is a key strength of this study, because alternative measurement approaches, such as using children’s self-report of their intake or parental proxies, are subject to substantial measurement error (29). Another strength is the use of within-child, fixed-effects regression models. Typical between-person regression models in observational studies cannot eliminate the possibility of confounding resulting from unmeasured differences between participants. Using participants as their own controls, however, renders time-stable confounders moot, whether measured or not (16). This approach was particularly helpful given that we could not measure several relevant child characteristics, such as socioeconomic status and BMI, which could have been confounders in a between-participants model. A general limitation of this approach is that it cannot eliminate the influence of time-varying confounders. It is possible, for example, that children who were more physically active on one day than on another were also more likely to bring a nonprogram snack to school and therefore consume more calories. However, given that a 10-year-old boy would need to walk for more than an hour to burn the excess 114.7 kcal of energy found in this study to be associated with a nonprogram snack (18), and given that overall child activity levels tend to be low, confounding by physical activity levels seems unlikely to explain the magnitude of the effect observed here.

Although this study found that consuming nonprogram snacks results in higher intake of total calories and unhealthy foods during the snack period, a key limitation of our study was our inability to assess the effect of nonprogram snacks on overall daily calorie intake. Children who did not have a nonprogram snack after school may have consumed more food at other points in the day. Our sample was limited to children who attended after-school programs in Boston and who were enrolled in the OSNAP trial and thus may not be generalizable to children who attend after-school programs in other locations or have different sociodemographic characteristics. Additionally, the effect of nonprogram snacks may be different in after-school programs where no snack is provided or where program-provided snacks are not held to any standards. Further research in other community settings that also evaluates the impact that nonprogram snacks have on total daily calorie intake is warranted, as is research on the role that policies and practices play in limiting or improving nonprogram snacks.

This study demonstrates that children who bring snacks to after-school programs that provide snacks and participate in federal snack programs may consume more calories (nearly double) and more servings of sugary foods and beverages than children who do not bring their own snacks. Allowing nonprogram snacks in after-school settings may increase children’s exposure to unhealthy foods and put them at risk for substantially increased intake of calories and sugary and salty foods. Limiting the availability of nonprogram snacks or setting standards for their nutritional content may contribute to healthier snack consumption among children who attend after-school programs in the United States.
